# Prognostic Value and Immune Infiltration Analysis of Nuclear Factor Erythroid-2 Family Members in Ovarian Cancer

**DOI:** 10.1155/2022/8672258

**Published:** 2022-01-11

**Authors:** Rui Dou, Xiong Wang, Jin Zhang

**Affiliations:** ^1^Department of Blood Transfusion, Henan Provincial People's Hospital, People's Hospital of Zhengzhou University, Zhengzhou 450003, China; ^2^Department of Laboratory Medicine, Tongji Hospital, Tongji Medical College, Huazhong University of Science and Technology, Wuhan 430030, China; ^3^Department of Public Health, Tongji Hospital, Tongji Medical College, Huazhong University of Science and Technology, Wuhan 430030, China

## Abstract

Ovarian cancer (OC) often presents at an advanced stage and is still one of the most frequent causes of gynecological cancer-related mortality worldwide. The nuclear factor erythroid-2 (NFE2) transcription factors include nuclear factor, erythroid 2 like 1 (NFE2L1), NFE2L2, and NFE2L3. NFE2 members bind to the antioxidant-response element (ARE) region and activate the expression of targeted genes. The distinct functions of NFE2 members in OC remain poorly elucidated. Several online bioinformatics databases were applied to determine gene expression, prognosis, mutations, and immune infiltration correlation in OC patients. NFE2L1 and NFE2L2 were decreased in OC, whereas NFE2L3 was increased. NFE2L2 and NFE2L3 were significantly correlated with the clinical stages of OC. High NFE2L1 level was significantly associated with short progression-free survival (PFS) in patients with OC (HR = 1.18, *P* = 0.021), while high NFE2L2 expression strongly correlated with long PFS (HR = 0.77, *P* = 0.00067). High NFE2L3 expression was associated with better overall survival and postprogression survival in OC. Functional analysis showed that NFE2 members mainly focused on transcription coactivator activities. Genetic alterations of NFE2 members were found in 13% of OC patients, and amplification ranked the top. The expression of NFE2 members was significantly correlated with immune infiltration of CD4+ T cells, CD8+ T cells, B cells, macrophages, and neutrophils in OC. Our study provides novel insights into the roles and prognostic potential of NFE2 family members in OC.

## 1. Introduction

Ovarian cancer (OC) ranks the third most common gynecologic malignancy but is the most lethal among these cancers, despite the fact that endometrial cancer has higher rate of incidence [1]. More than 300, 000 new cases of OC with more than 190, 000 deaths were expected in 2020 worldwide, and most cases are diagnosed at advanced stages. Primary cytoreduction combined with chemotherapy is the first-line treatment for OC patients in advanced stage [2]. Risk factors for OC include *BRCA1* and/or *BRCA2* mutation, endometriosis, increasing age, infertility, polycystic ovarian syndrome, and use of an intrauterine device [3]. Exploring novel biomarkers for tumorigenesis and progression of OC is necessary.

The nuclear factor erythroid-2 (NFE2) transcription factors (TFs) include nuclear factor, erythroid 2 like 1 (NFE2L1), NFE2L2, and NFE2L3 [4]. All three NFE2 TFs bind to the ARE (antioxidant-response element) region and activate the expression of targeted genes, including those encoding ferritin, heme oxygenase 1, and metallothionein [5, 6]. The NFE2 TFs are involved in tumorigenesis and therapeutic resistance in some cancers [7, 8].

We studied the prognostic significance and immune infiltration association of the NFE2 TFs in OC based on numerous databases.

## 2. Materials and Methods

### 2.1. Expression of NFE2 Transcription Factors in OC

GEPIA is a recently developed analytical tool for estimating expression level based on RNA sequencing (RNA-Seq) of 8587 normal and 9,736 tumor samples in GTEx and TCGA databases (http://gepia2.cancer-pku.cn/#index) [9]. GEPIA was applied to perform differential expression analysis.

### 2.2. Prognostic Value of NFE2 Transcription Factors in OC

KM plotter was widely used to screen prognostic and survival biomarkers according to gene expression from 20 different cancers including 11k samples (http://kmplot.com/analysis/) [10]. NFE2 proteins' prognostic values for progression-free survival (PFS), postprogression survival (PPS), and overall survival (OS) were assessed. *P* < 0.05 was considered statistically significant.

### 2.3. cBioPortal

cBioPortal contains multidimensional cancer genomics data from 20 different cancer studies including over 5,000 tumors. Gene alterations within the three genes were obtained, including missense mutations, amplifications, and deep deletions (http://www.cbioportal.org/) [11, 12].

### 2.4. STRING

STRING is used to achieve a comprehensive protein-protein interaction (PPI) network with a unique set of computational predictions (https://string-db.org/) [13]. PPI network was applied to clarify mechanisms relevant to the tumorigenesis of OC.

### 2.5. GeneMANIA

GeneMANIA is an online server to explore the association between genes concerning the colocalization, coexpression, pathway, physical interactions, and shared protein domains (http://genemania.org/) [14].

### 2.6. TIMER 2

TIMER 2 is used to evaluate the correlation of gene expression and immune infiltration of diverse immune cells from 32 cancers using Spearman correlation analysis (http://timer.cistrome.org/) [15]. In this study, scatterplots were generated with tumor purity and immune infiltration of main immune cells (B cells, CD4+ T cells, CD8+ T cells, neutrophils, macrophages, and dendritic cells). Correlation was analyzed using Pearson's correlation.

## 3. Results

### 3.1. Differential Expression of the NFE2 Members in OC

**Figure 1:**
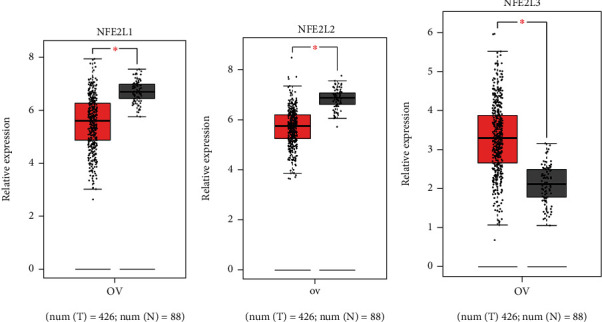
Differential expression of the NFE2 members in OC patients. Expression of the nuclear factor erythroid-2 (NFE2) members in ovarian cancer (OC) was analyzed using GEPIA. Nuclear factor, erythroid 2 like 1 (NFE2L1) and NFE2L2 were lower in OC (a, b), while NFE2L3 was higher in OC (b). ^∗^*P* < 0.05.

GEPIA dataset was selected to analyze the expression levels of NFE2 members in OC. The results indicated that NFE2L1 and NFE2L2 were decreased in OC, while NFE2L3 was overexpressed in OC (Figures 1(a)–1(c)).

### 3.2. Association between NFE2 Member Expression and Clinical Stages in OC

**Figure 2:**
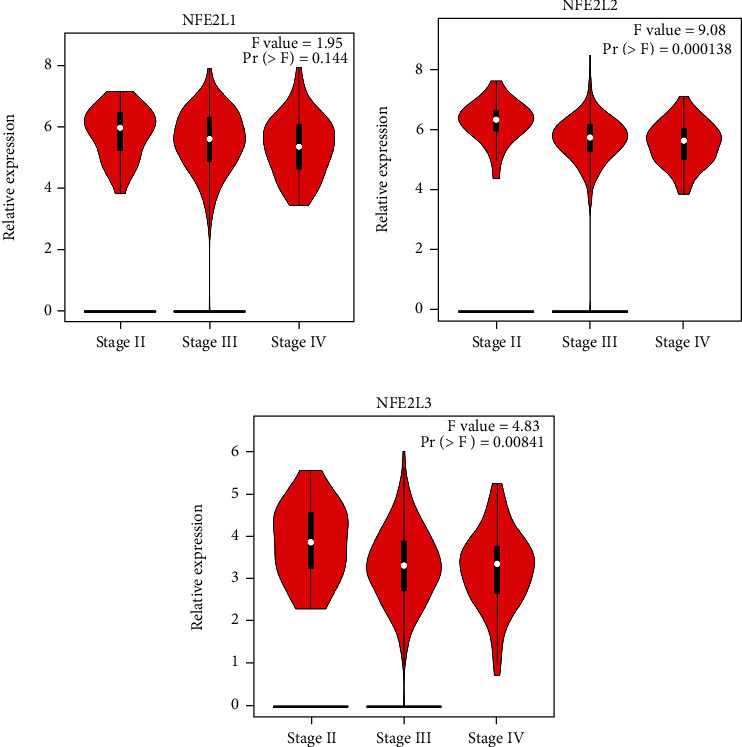
Association between the NFE2 member expression and clinicopathological stages in OC. Correlation between the NFE2 member expression and tumor stages in OC patients was assessed using GEPIA.

The correlation between expression levels of NFE2 members and the clinical stage in OC was analyzed with GEPIA. The NFE2L2 and NFE2L3 showed lower expression in advanced stage than the earlier stage, whereas NFE2L1 was similar among groups (Figures 2(a)–2(c)).

### 3.3. Prognostic Analysis of NFE2 Members in OC Patients

**Figure 3:**
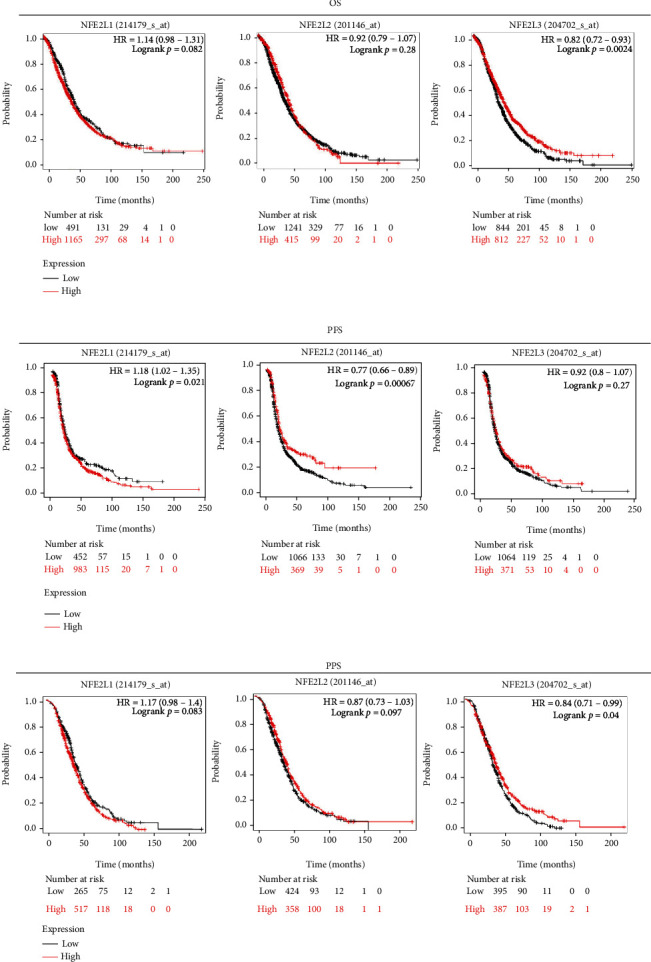
Prognostic analysis of the NFE2 members in OC patients: (a) overall survival (OS); (b) progression-free survival (PFS); (c) postprogression survival (PPS).

Prognostic analysis of the NFE2 members in OC patients was investigated using KM plotter. Higher NFE2L1 level was remarkably associated with shorter PFS in OC patients (HR = 1.18, *P* = 0.021), while higher NFE2L2 strongly correlated with better PFS (HR = 0.77, *P* = 0.00067). Higher NFE2L3 significantly correlated with better PPS and OS in OC patients (Figures 3(a)–3(c)).

### 3.4. Genetic Mutation and Interaction Analysis of NFE2 Members in OC Patients

**Figure 4:**
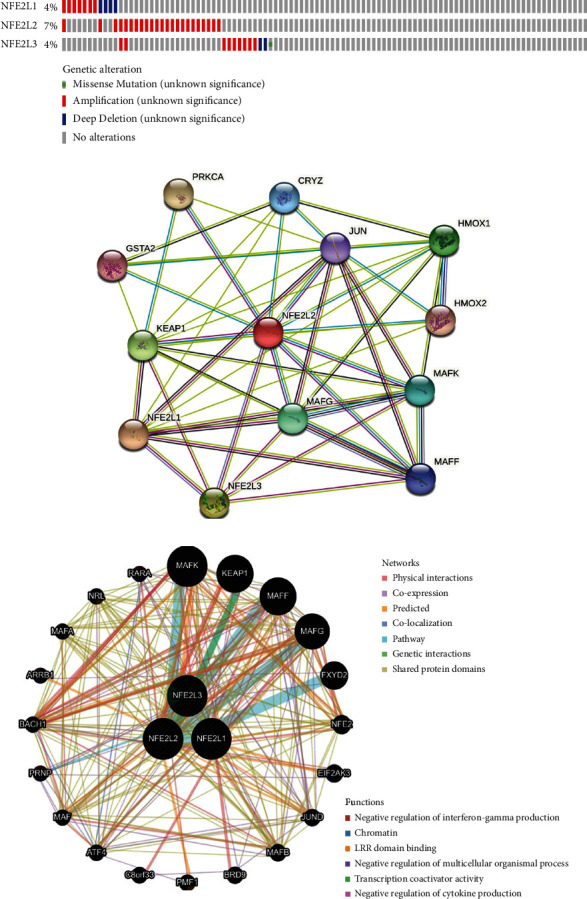
Genetic alteration and interaction analyses of NFE2 members in OC patients: (a) genetic alternation frequencies of the NFE2 members in OC patients were analyzed using cBioPortal. PPI network of different expressed NFE2 proteins was analyzed using STRING (b) and GeneMANIA (c).

Genetic mutations of the NFE2 members in OC patients were analyzed with cBioPortal. Overall, mutations of the NFE2 members were found in 41 samples of 311 OC patients, accounting for 13%. Moreover, NFE2L1, NFE2L2, and NFE2L3 were altered in 4%, 7%, and 4% of OC patients, respectively (Figure 4(a)).

The PPI network of the differentially expressed NFE2 proteins was conducted with STRING and GeneMANIA (Figures 4(b) and 4(c)). The greatest associated genes include MAFK, KEAP1, MAFF, and MAFG. Their main biological process was primarily related to transcription coactivator activity.

### 3.5. Correlation between NFE2 Member Level and Immune Infiltration in OC

**Figure 5:**
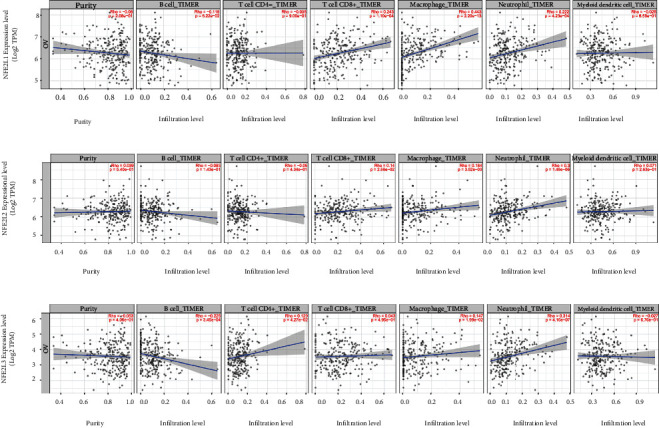
Association of NFE2 member expression with immune infiltration in OC: (a) NFE2L1; (b) NFE2L2; (c) NFE2L3.

The correlation between NFE2 family members and immune infiltration of the main immune cells in OC was determined using TIMER 2. NFE2L1 and NFE2L2 were significantly positively correlated with infiltration of CD8+ T cell, macrophage, and neutrophil cell in OC (Figures 5(a) and 5(b)). NFE2L3 expression was significantly positively associated with infiltration of CD4+ T cell, macrophage, and neutrophil cell, while it is negatively correlated with B cells (Figure 5(c)).

## 4. Discussion

OC is typically discovered at advanced stages and is still one of the most frequent causes of gynecological cancer-related mortality worldwide. Moreover, the current screening strategy fails to reduce deaths [16]. Treatments for newly diagnosed OC patients combine surgical cytoreduction and chemotherapy; immunological therapies are being tested currently [17]. BRCA1 and BRCA1 have been considered as genetic risk factors for OC. Recent progress in RNA-Seq has broadened the knowledge of the molecular pathogenesis of OC and helps identify molecular biomarkers for OC early detection and prognosis prediction [18, 19]. TFs regulate cellular process and function via activating or repressing target gene expression. Dysregulation of TFs may contribute to tumorigenesis [20]. The NFE2 family consists of NFE2L1, NFE2L2, and NFE2L3, and we explored their prognostic value and immune infiltration correlation in OC.

NFE2L1 is a critical proteasome regulator in cancer cells for the maintenance of their basal proteasome activities via activating proteasome-related genes, and NFE2L1 knock-out mice suffered embryonic lethality [21]. NFE2L1 showed high expression in esophageal carcinoma, lymphoid neoplasm, diffuse large B-cell lymphoma, pancreatic adenocarcinoma, and thymoma, while low expression in uterine corpus endometrial carcinoma, ovarian serous cystadenocarcinoma, and uterine carcinosarcoma analyzed by GEPIA web server (http://gepia.cancer-pku.cn/detail.php?gene=NFE2L1). Silencing of NFE2L1 induced aggressiveness and chemoresistance in pancreatic endocrine tumors, indicating a tumor-suppressive role of NFE2L1 [22]. In our study, NFE2L1 was decreased in OC and its expression was not correlated with different stages. High expression of NFE2L1 was correlated with short PFS. These results suggest prognostic biomarker potential of NFE2L1 in OC.

NFE2L2 is a Cap'n' collar (CNC), leucine zipper (bZIP) TF and expressed in all cell types. NFE2L2 is the main regulator in cellular antioxidant response. NFE2L2-targeted genes regulate drug metabolism; redox homeostasis; iron, energetic, and amino acid metabolism; proteasomal degradation; proliferation; survival; and mitochondrial physiology [23]. NFE2L2 was mainly considered as oncogene, which promoted metastasis, progression, and resistance to chemo- and radiotherapy. However, tumor-suppressive effects of NFE2L2 have also been observed [24]. We found that NFE2L2 was decreased in OC and negatively associated with clinical stage in OC. High expression of NFE2L2 was correlated with long PFS. These results indicate a tumor suppressive role of NFE2L2 in OC.

NFE2L3 is the homologous gene of NFE2L1 [25]. NFE2L3 could suppress NFE2L1 translation via suppressing the polysome formation on NFE2L1 mRNA by targeting CPEB3 [26]. NFE2L3 functioned as an oncogene in colon cancer by suppressing the expression of DUX4, which functioned as a direct inhibitor of CDK1 [27]. High expression of NFE2L3 predicted poor prognosis of pancreatic cancer [28]. NFE2L3 promoted cell proliferation and metastasis of hepatocellular carcinoma through activation of the Wnt/*β*-catenin pathway [29]. NFE2L3 was increased in OC and negatively correlated with clinical stage. Higher NFE2L3 was correlated with longer PPS and OS in OC, indicating a prognostic potential in OC.

Immune cells located in tumor microenvironment (TME) performed tumor-promoting or tumor-suppressing potentials, and TME could affect tumor progression and recurrence. TME has predictive potential of immunotherapy reactivity and clinical outcome [30]. The expression of NFE2 members was correlated with infiltration of CD4+ T cells, CD8+ T cells, B cells, macrophages, and neutrophils, suggesting predictive potential of NFE2 family members in OC for immune checkpoint blockade therapy. NFE2 family members and their interacting genes strongly correlated with transcription activity as illustrated by GeneMANIA and STRING database.

There were some limitations. Our study is based on data retrieved from online databases. Only gene expression was considered, and the protein level was not discussed. Potential mechanisms require further in vitro and in vivo cellular experiments and clinical studies to further validate our findings.

In conclusion, we studied the prognostic significance and immune infiltration association of NFE2 family members in OC through bioinformatics analysis. NFE2L1 and NFE2L2 were significantly decreased in OC, while the expression of NFE2L3 was increased. Higher NFE2L1 was significantly correlated with shorter PFS in OC, while higher NFE2L2 was strongly correlated with better PFS. NFE2L3 was correlated with long PPS and OS. The NFE2 family members were significantly correlated with immune infiltration. Our study provides a novel insight into the roles and prognostic potential of NFE2 family members in OC.

## 5. Conclusions

The expression of NFE2L1 and NFE2L2 was decreased in OC, while NFE2L3 was increased in OC. NFE2L1 was significantly correlated with short PFS in OC patients, while higher NFE2L2 level was remarkably correlated with better PFS. Moreover, NFE2L3 was correlated with long PPS and OS. The NFE2 family was also strongly correlated with immune infiltration. Our study provides a novel perspective on the distinct roles and prognostic potential of NFE2 family members in OC.

## Data Availability

All datasets generated for this study are included in the article.

## Abbreviations

OC: Ovarian cancer
NFE2: Nuclear factor erythroid-2
TF: Transcription factor
NFE2L1: Nuclear factor, erythroid 2 like 1
NFE2L2: Nuclear factor, erythroid 2 like 2
NFE2L3: Nuclear factor, erythroid 2 like 3
ARE: Antioxidant-response element
GTEx: Genotype-Tissue Expression dataset projects
TCGA: The Cancer Genome Atlas
OS: Overall survival
PFS: Progression-free survival
PPS: Postprogression survival
PPI: Protein-protein interaction
CPEB3: Cytoplasmic polyadenylation element-binding protein 3
TME: Tumor microenvironment.
